# Complete chloroplast genome of the wild Japanese Mountain cherry (*Prunus jamasakura*, Rosaceae)

**DOI:** 10.1080/23802359.2019.1699463

**Published:** 2019-12-13

**Authors:** Xian-Gui Yi, Jie Chen, Meng Li, Hong Zhu, Zhong-Shuai Sun, Toshio Katsuki, Xian-Rong Wang

**Affiliations:** a Co-Innovation Center for the Sustainable Forestry in Southern China; Cerasus Research Center; College of Biology and the Environment, Nanjing Forestry University, Nanjing, China;; b Zhejiang Provincial Key Laboratory of Plant Evolutionary Ecology and Conservation, Taizhou University, Taizhou, China;; c Tama Forest Science Garden, Forestry and Forest Products Research Institute, Hachioji, Japan

**Keywords:** *Prunus jamasakura*, *Prunus sensu lato*, chloroplast genome, phylogenomics

## Abstract

*Prunus jamasakura* is a species of Prunus native to eastern Asia. We determined the first complete chloroplast genome of *Prunus jamasakura* using genome skimming approach. The cp genome was 157,905 bp long, with a large single-copy region (LSC) of 85,910 bp and a small single-copy region (SSC) of 19,123 bp separated by a pair of inverted repeats (IRs) of 26,436 bp. It encodes 129 genes, including 84 protein-coding genes, 37 tRNA genes, and 8 ribosomal RNA genes. We also reconstructed the phylogeny of *Prunus sensu lato* using maximum likelihood (ML) method, including our data and previously reported cp genomes of related taxa. The phylogenetic analysis indicated that *P. jamasakura* is closely related with *Prunus speciosa*.

*P. jamasakura* Siebold ex Koidz., Japanese mountain cherry, is one of the trees typical of the secondary forests on the mountains of southern and western Japan (Kuitert and Peterse 1999; Ohba 2001). The classification of the *Prunus sensu lato* (Rosaceae) has long been problematic; phylogenetic studies using a limited set of markers have often not been able to fully resolve relationships within this genus, indicating that a higher number of molecular characters are required for an improved understanding of relationships within this group (Shi et al. 2013; Chin et al. 2014). By taking advantages of next-generation sequencing technologies that efficiently provide the chloroplast (cp) genomic resources of our interested species, we can rapidly access the abundant genetic information for phylogenetic research and conservation genetics (Liu et al. 2017, 2018). Therefore, we sequenced the whole chloroplast genome of *P. jamasakura* to elucidate its phylogenetic relationship with other *Prunus sensu lato*.

Total genomic DNA was extracted from silica-dried leaves collected from Botanical Gardens Faculty of Science Osaka City University (Katano, Osaka, Japan) using a modified CTAB method (Doyle and Doyle 1987). The voucher specimen (Sun1704035) was collected and deposited in the Herbarium of Zhejiang Academy of Forestry. DNA libraries preparation and pair-end 125 bp read length sequencing were performed on the Illumina HiSeq 2500 platform. About 10.8 Gb of raw data were trimmed and assembled into contigs using CLC Genomics Workbench 8. All the contigs were then mapped to the reference cp genome of *Prunus speciosa* (Koidz.) Nakai (MH998233; Sun et al. 2019) using BLAST (NCBI BLAST v2.2.31) search and the draft *cp* genome of *P. jamasakura* was constructed by connecting overlapping terminal sequences in Geneious R11 software (Biomatters Ltd., Auckland, New Zealand). Gene annotation was performed via the online program Dual Organellar Genome Annotator (DOGMA; Wyman et al. 2004).

The complete cp genome of *P. jamasakura* (GenBank accession MN652612) was 157,905 bp long consisting of a pair of inverted repeat regions (IRs with 26,436 bp) divided by two single-copy regions (LSC with 85,910 bp; SSC with 19,123 bp). The overall GC contents of the total length, LSC, SSC, and IR regions were 36.7%, 34.6%, 30.2% and 42.5%, respectively. The genome contained a total of 129 genes, including 84 protein-coding genes, 37 tRNA genes and 8 rRNA genes.

We used a total of 21 additional complete cp genomes of the *Prunus sensu lato* species to clarify the phylogenetic position of *P. jamasakura*. *Prunus serotina* Ehrh. (NC036133) and *P. padus* L. (NC026982) in Subg. *Padus* were used as the outgroup. We reconstructed a phylogeny employing the GTR + G model and 1000 bootstrap replicates under the maximum-likelihood (ML) inference in RAxML-HPC v.8.2.10 on the CIPRES cluster (Miller et al. 2010). The ML tree (Figure 1) was consistent with the most recent phylogenetic study on *Prunus sensu lato* (Shi et al. 2013; Chin et al. 2014). *P. jamasakura* exhibited the closest relationship with *P. speciosa*.

**Figure 1.**
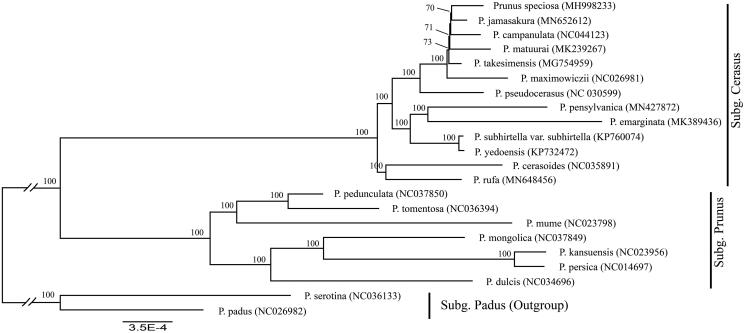
Phylogenetic tree reconstruction of 22 taxa of *Prunus sensu lato* using ML method. Relative branch lengths are indicated. Numbers near the nodes represent ML bootstrap value. The scientific names of some species are debated.
